# ANN-Based Discernment of Septic and Inflammatory Synovial Fluid: A Novel Method Using Viscosity Data from a QCR Sensor

**DOI:** 10.3390/s22239413

**Published:** 2022-12-02

**Authors:** Andrés Miranda-Martínez, Berta Sufrate-Vergara, Belén Fernández-Puntero, María José Alcaide-Martin, Antonio Buño-Soto, José Javier Serrano-Olmedo

**Affiliations:** 1 Centre for Biomedical Technology (CTB), Universidad Politécnica de Madrid (UPM), 28040 Madrid, Spain; 2 Department of Clinical Analysis-Emergency, Hospital Universitario La Paz (HULP), 28046 Madrid, Spain; 3 Networking Research Center of Bioengineering, Biomaterials and Nanomedicine (CIBER-BBN), Universidad Politécnica de Madrid (UPM), 28040 Madrid, Spain

**Keywords:** quartz crystal resonator, synovial fluid, septic synovial fluid, inflammatory synovial fluid, Artificial Neural Networks

## Abstract

The synovial fluid (SF) analysis involves a series of chemical and physical studies that allow opportune diagnosing of septic, inflammatory, non-inflammatory, and other pathologies in joints. Among the variety of analyses to be performed on the synovial fluid, the study of viscosity can help distinguish between these conditions, since this property is affected in pathological cases. The problem with viscosity measurement is that it usually requires a large sample volume, or the necessary instrumentation is bulky and expensive. This study compares the viscosity of normal synovial fluid samples with samples with infectious and inflammatory pathologies and classifies them using an ANN (Artificial Neural Network). For this purpose, a low-cost, portable QCR-based sensor (10 MHz) was used to measure the viscous responses of the samples by obtaining three parameters: Δf, ΔΓ (parameters associated with the viscoelastic properties of the fluid), and viscosity calculation. These values were used to train the algorithm. Different versions of the ANN were compared, along with other models, such as SVM and random forest. Thirty-three samples of SF were analyzed. Our study suggests that the viscosity characterized by our sensor can help distinguish infectious synovial fluid, and that implementation of ANN improves the accuracy of synovial fluid classification.

## 1. Introduction

Synovial fluid (SF) is a viscous liquid located in the joints whose primary functions are twofold. The first is the joint’s mechanical function, which involves lubricating the articular surface and cushioning movements. The second one is to contribute to the nutrition of the articular cartilage by acting as a nutrient transport medium. It is composed of dialysate of plasma and a high content of hyaluronic acid (HA), which is responsible for its viscosity [1,2].

The analysis of SF begins with the extraction of the sample (by arthrocentesis), which involves a joint puncture. Then, the sample is collected in tubes containing anticoagulants such as EDTA (ethylenediaminetetraacetic acid) and lithium heparin [1,3,4]. Regarding volume, the maximum amount obtained from a normal joint is between 0.1 and 3.5 mL. The knee can have up to 4 mL. The volume required depends on the analysis (and may vary between laboratories). For example, for an accurate cell count, approximately 1 mL is required; 2 to 3 mL is an adequate volume to perform the complete tests needed. If a low-volume sample is obtained, it should be sent for the analysis of crystals and culture, which are more useful for the diagnostic [1,4,5].

To determine the viscosity (η), it was usual to observe the stranding, i.e., to measure the “thread” formed by the liquid when extended. This can be done by placing the sample drop on a slide and lifting it with a spatula or using the thumb and forefinger to spread it out. The “thread” may measure between 3 and 6 cm for a healthy fluid. SF with poor viscosity will form a “thread” of less than 3 cm [1,4,6]. Being a subjective method, as it depends on the operator’s skills and experience, its use has been decreasing. As an objective assessment of viscosity, it is possible to use a viscometer or rheometer; however, they usually require more sample volume than is available or are expensive and large.

HA concentration determines the SF’s viscoelastic properties. Arthritic diseases are associated with the reduction of HA [4,5,7,8]. In healthy SF, the concentration is around 3.5 mg/mL, whereas in osteoarthritis (OA), the HA concentration decreases to 1.3 mg/mL, and in rheumatoid arthritis (RA) to approximately 0.84 mg/mL [9]. This reduction in HA leads to a decrease in SF viscosity [5]. Joint diseases increase the risk of septic arthritis, which requires prompt diagnosis, as it is essential to provide the treatment as soon as possible [10].

On the other hand, the feasibility of using quartz crystal resonators (QCRs) as sensors to detect viscosity changes has been demonstrated [11,12,13]. These sensors are better known as quartz crystal microbalances (QCMs) [14,15,16]. They have also been used to detect specific agents and identify diseases such as influenza [17,18,19], malaria [20,21], human immunodeficiency virus (HIV) [20,22], tuberculosis [23,24,25], Alzheimer’s [26,27], and breast cancer [28]. In addition, experiments was conducted in [29] to observe the responses of these sensors when measuring blood, hoping to help in cardiovascular disease prevention.

Within the ViSQCT project of the Universidad Politécnica de Madrid (UPM), we developed a prototype sensor whose operation is based on the use of QCR. Its use in characterizing the viscosity of hydrogel formation has been previously demonstrated [30]. In a previous study [11], its operation was detailed, and its usefulness in measuring samples of artificial synovial fluid was tested. The sensor’s objective is to measure the viscosity of a fluid with a small sample volume and to use this information to discriminate between pathologies and thus provide a timely diagnosis.

As part of the development of the device, an Artificial Neural Network (ANN) was implemented to optimize the classification of SF samples. ANNs have made inroads in biomedical engineering thanks to their ability to find relationships between data for prediction or classification [31,32]. Some examples of their use in biomedical applications can be seen in [31,32,33,34]. Additionally, their use with QCM sensors can be seen in the works [35,36,37]. In this work, we show the application of and comparison between parameters of an ANN to classify synovial fluid as inflammatory or infectious. This was done with data obtained from measurements performed with the QCR-based sensor. As a comparison, two other classification models were trained: support vector machine (SVM) and random forest (RF). SVM models are related to multilayer ANNs, and their operation is based on establishing a boundary (margin) that separates the two classes [38]. On the other hand, RF is an ensemble learning technique that has gained popularity due to its great capacity for classification [39].

The main contributions of the paper can be summarized as follows:

- It is demonstrated that the ViSQCT sensor effectively measures the viscosity change in low-volume samples of SF.
- A complete methodology is proposed to differentiate between inflammatory and infectious SF.
- We show that using classification models such as ANN improves the methodology by increasing classification accuracy.
- We compare the performance of the methodology and the system when using SF samples stored in two types of tubes (tubes with EDTA and tubes with lithium heparin) and evaluate their influences on making an accurate differentiation.

The present work shows the use of a portable and low-cost (less than EUR 200) QCR-based sensor named “ViSQCT” (developed in-house at the UPM) which allows the characterization of the viscosity of a small volume sample (few microliters) to classify between inflammatory and septic SF. The ethics committees of both the hospital and the university approved this work.

## 2. Materials and Methods

### 2.1. Synovial Fluid Samples

The Hospital Universitario La Paz (Madrid, Spain) provided the SF samples. We used the remnants of samples sent to the Emergency Laboratory collected from July 2021 to September 2021 to be analyzed for diagnosis. Thirty-three samples from different patients were provided in tubes with EDTA, of which 28 were additionally submitted in tubes with lithium heparin. Based on clinical and laboratory parameters, the samples (Table 1) were classified into two main groups: inflammatory pathology (rheumatoid arthritis, gout, psoriatic arthritis, etc.) and infectious pathology (septic arthritis and prosthetic infections). Additionally, the data of white blood cells (WBC/mm${}^{3}$), neutrophils (%), glucose (mg/dL), and proteins (g/dL) of the fluids were proportioned.

### 2.2. Sensor

The sensor used has been developed as part of the ViSQCT project of the Bioinstrumentation and Nanomedicine Laboratory (LBN) of the UPM. A complete description can be found in [11]. Its basis of operation is the use of the series resonance frequency ($f_{s}$) of the QCR. Resonance frequency obtention is achieved by exciting the crystal with a frequency sweep near the fundamental resonance frequency and obtaining the conductance curve. With this, we locate the frequency where the maximum conductance is. The frequency shift (Equation (1)) is obtained by doing this process in air (without sample) and then with the sample deposited on the crystal. The Kanazawa relationship gave the connection between the frequency shift and the density-viscosity product of the fluid in contact with the crystal (Equation (2)) [40]. The half-bandwidth at half-maximum (Γ) is also acquired from the conductance curve, and like the resonance frequency case, the shift ΔΓ is obtained. This parameter is related to the energy transferred from the crystal to the sample over time and can provide information on the viscoelastic properties of the sample [41].

(1) $$\Delta f=f_{s}-f_{0}$$

(2) $$\Delta f=-\sqrt{n}f_{0}^{3/2}\sqrt{\frac{\rho_{L}\eta_{L}}{\pi \rho_{q}G_{q}}},$$

where $\rho_{q}=2.648$ gcm${}^{-3}$ and $G_{q}$ = 2.947 ×$10^{10}$ Nm${}^{-2}$ are the specific density and the shear modulus of quartz, respectively; $f_{0}$ is the fundamental resonance frequency of the quartz; $f_{s}$ is the series resonance frequency of the crystal loaded; $\rho_{L}$ is the fluid’s density; $\eta_{L}$ is the fluid’s viscosity; Δf is the frequency shift; and finally, n is the overtone number. In this work, the fundamental frequency of the crystal was used; thus, n was 1.

This work was performed using QCR with $f_{0}$ = 10 MHz, gold electrodes, 5 and 11 mm electrode dimensions, roughness < 1 nm, and mounted in HC-51 holder. The crystals were purchased from Krystaly (Hradec Králové, Czech Republic).

### 2.3. Experimental Set-Up

**Figure 1:**
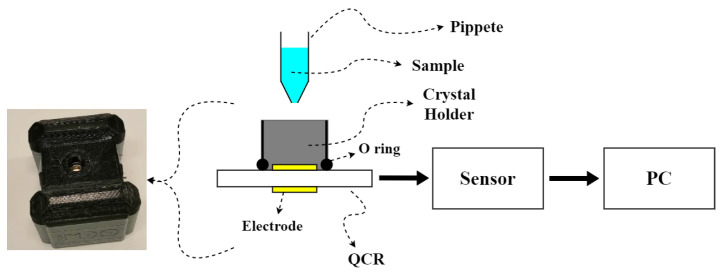
Experimental set-up.

The experimental setup is illustrated in Figure 1. The QCR was placed inside the holder cell where the liquid sample was dropped. The sample volume was 50 μL, since it was to cover the crystal’s surface entirely and not completely evaporate. Experiments were performed at room temperature. Each experiment was repeated three to five times. Each experiment lasted 5 min, wherein 50 measured points were obtained (1 point every 6 s). In this way, the dataset was formed. The parameters Δf, ΔΓ, and η obtained from Δf were measured. After each experiment, the crystal was cleaned using a 2% solution of sodium dodecyl sulfate, rinsed with distilled water, disinfected with 70% ethanol, and then rinsed again with distilled water. Finally, the electrode surface was dried with air.

### 2.4. Statistical Analysis

Statistical analysis was performed with SPSS, statistical software. Means are expressed as mean ± standard deviation. Mann–Whitney U was used for analytic comparison; *p*-values less than 0.05 were considered statistically significant. The predictive abilities regarding septic SF of Δf, ΔΓ, and η were expressed as the area under the receiver operating characteristic curve (AUC-ROC); AUC values are reported with their 95% confidence intervals (95% CI).

### 2.5. Artificial Neural Networks

**Figure 2:**
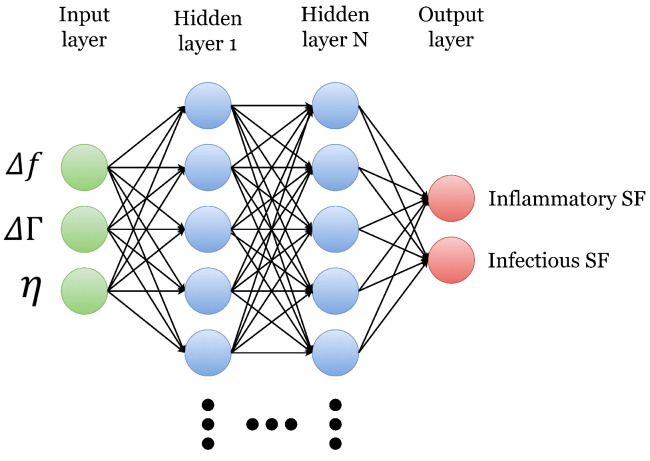
Artificial Neural Network model.

ANNs are a case of Artificial Intelligence (AI) that, based on examples, can induce concepts. They are data processing systems whose operation is based on the networks of neurons in the brain [31,32]. These tools help find relationships between data and also in classification and prediction. They can also improve their performances by using information obtained from previous tasks. The basic model of the ANN (known as the multilayer perceptron model) is shown in Figure 2. It comprises three layers: an input layer, an output layer, and hidden layers (HL). This model allows information to flow in one direction, from input to output, and is known as a feedforward neural network. This way, data will enter the network’s input nodes, then be processed in the hidden layers, and finally be delivered to the output layer [32].

**Figure 3:**
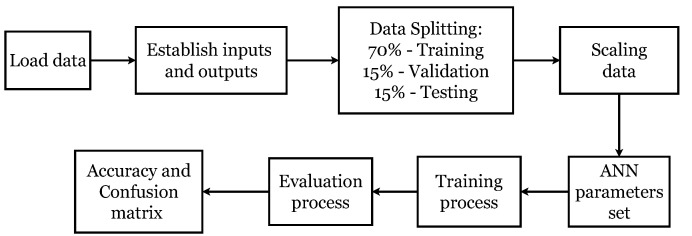
ANN algorithm for SF classification.

The ANN was applied using the algorithm illustrated in the diagram in Figure 3. As shown in Figure 2, the input data were the parameters Δf, ΔΓ, and η obtained with the sensor. The output values (or labels) were some the two possible diagnoses provided by the hospital (inflammatory and infectious SF). Having a larger amount of inflammatory SF samples (imbalanced data), the algorithm was tested using the imbalanced data and then with balanced data. The balanced data were obtained by randomly oversampling the septic SF data, thereby achieving the same data for both classifications.

**Figure 4:**
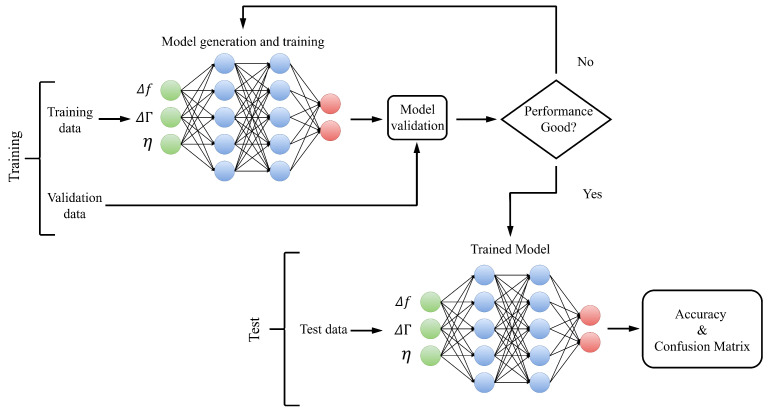
Training and test in the ANN model.

The dataset size was 4972 data for samples in tubes with EDTA and 5248 for samples in tubes with lithium heparin. After loading the input data, the data were randomly segmented for the training, validation, and test phases as 70, 15, and 15%. Thus, 70% of the dataset was used for training, 15% was used for validation, and the remaining 15% was isolated for testing with the trained model. This way, we had three datasets: training, validation, and test. The training dataset contained the examples used during the learning process and was used to adjust the parameters. A validation dataset was a set of examples used to adjust the hyperparameters. The test dataset was a separate dataset from the training dataset used to test the model after training. After the data splitting, the data were optimized by scaling them to a range of values between 0 and 1. A robust scaler was employed, which scales the information according to the quantile range, making it robust against outliers. Figure 4 shows the steps of the ANN model.

The accuracy value was obtained for each case to observe the algorithm’s performance. Accuracy is obtained from the fraction of the total number of correct predictions divided by the sum of all predictions (Equation (3)):

(3) $$Accuracy=\frac{\mathrm{Number}\;\mathrm{of}\;\mathrm{correct}\;\mathrm{predictions}}{\mathrm{Total}\;\mathrm{number}\;\mathrm{of}\;\mathrm{predictions}}=\frac{TP+TN}{TP+TN+FP+FN}$$

where TP = true positives, TN = true negatives, FP = false positives, and FN = false negatives.

Finally, to compare different ANN configurations, the HL of the networks were varied between 1 and 2 layers, and the number of training epochs among 100, 200, and 300. These configurations are shown in Table 2. Parameters such as the optimizer, activation function, and biases were left constant, since it is beyond the scope of this work to go into this topic in more detail. A more extensive study with a more significant number of configurations is possible, as the field of ANN is vast; however, this is beyond the intended scope of this paper.

All algorithms were developed using the Keras and sci-kit learn libraries in Python. We used a linear kernel for SVM, c = 1, loss = “squared hinge.” For RF, we used 2171 trees, minimum sample split = 2, maximum depth = 200, and criterion = “gini.” The hyperparameters for the RF model were established by a previous exploration (tuning) with a grid search. For this, a range of values was defined, and a search algorithm performed a random search of those values and found the best one. The default setting was used for the SVM model while adding the “squared hinge loss,” which is common for binary classifications [42]. For the SVM and RF cases, 85% of the dataset was used for training, and 15% as test set.

## 3. Results

Concerning the parameters measured with the sensor, there were no statistically significant differences between the mean values of η and Δf for the case of SF contained in tubes with EDTA. However, in this case, a statistically significant difference was observed for ΔΓ. When comparing both samples of SF collected in tubes with lithium heparin, there were significant differences in the mean values of Δf and ΔΓ, but not for η (Table 3 and Table 4). When looking at the differences between the data provided by the hospital, WBC is shown to have the most consistent data—significant differences in both cases.

**Figure 5:**
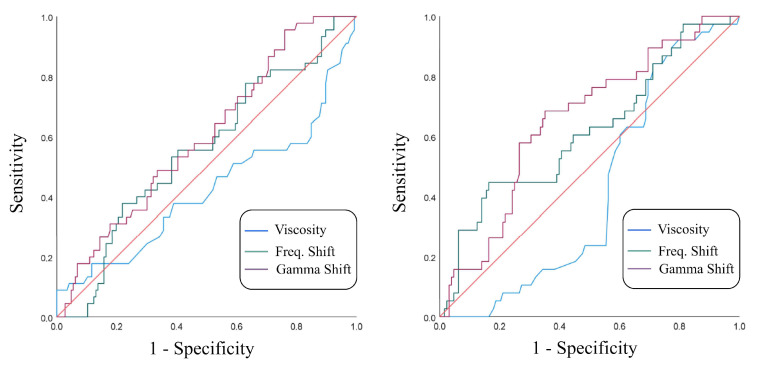
ROC curves for parameters measured with the sensor for: SF in tubes with EDTA (**left**) and SF in tubes with lithium heparin (**right**).

The predictive ability of each parameter is shown in Figure 5 (ROC curve) and Table 5 and Table 6, which illustrates the area value under the ROC curve (AUC), confidence interval (CI), and standard error (SE). Shown for reference are the WBC, serum procalcitonin (PCT), and SF PCT parameters obtained in a different study [10].

In Figure 5, we can see that the viscosity calculation obtained does not discriminate the infectious SF well. On the other hand, Δf and ΔΓ had better results on the samples contained in tubes with lithium heparin, although they did not become a test that stands out.

The obtained parameters showed slightly better performance in samples stored in tubes with lithium heparin; nevertheless, they are far from being decisive for classification. One study [10] showed that procalcitonin (PCT) is used as a marker to discriminate infectious SF. The study showed that the WBC value is the most accurate at the time of distinguishing infectious SF (AUC = 1). When evaluating the value of PCT in serum and PCT in SF, they showed that PCT in serum was better (AUC = 0.82) than PCT in SF (AUC = 0.65). This last value is comparable with the Δf (AUC = 0.61) and ΔΓ (AUC = 0.65) obtained in this work (tubes with lithium heparin).

When observing the results, it is noticeable that the SF samples contained in tubes with lithium heparin showed higher Δf, ΔΓ, and η values. This may have been due to a change in the sample’s viscosity generated by the type of anticoagulant in the tube. Studies show that lithium heparin can lead to accumulations of white blood cells, which may explain this phenomenon [43,44].

**Figure 6:**
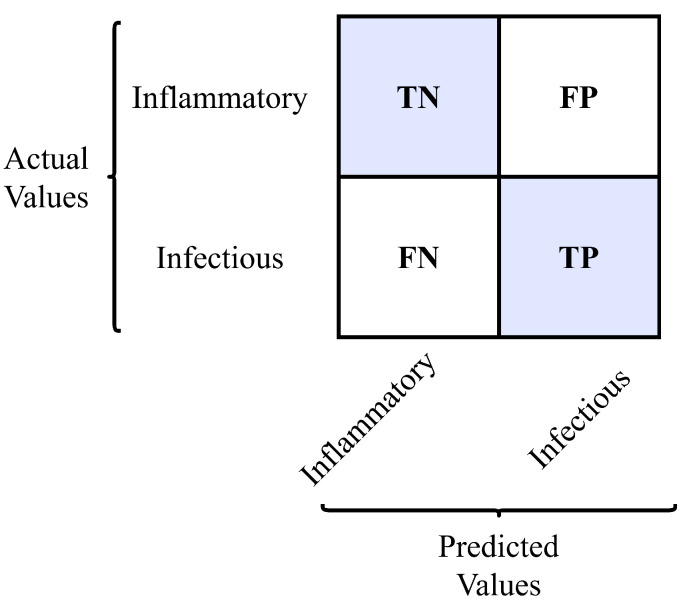
Confusion matrix for SF classification.

Based on the low performance of each parameter individually in differentiating SF precisely, there was interest in testing AI algorithms to see if they can help better classify the samples. When classifying by ANN, six scenarios were analyzed for each container case of the SF samples. Table 7 shows the accuracy values obtained in each case. In this article, the confusion matrix for each scenario is distributed as follows: TP: the real classification was inflammatory SF, and the prediction was made correctly. TN: the real classification was infectious SF, and the prediction was made correctly. FP: the real classification was infectious SF, and the prediction was made incorrectly. FN: the real classification was inflammatory SF, and the prediction was made incorrectly. This can be best seen in Figure 6.

When viewing the accuracy values obtained in Table 7, it is clear that samples contained in lithium heparin tubes performed better in the classification for both cases. Considering the imbalanced data as input elements to the ANN, increasing the number of epochs also increased the accuracy. By increasing the number of hidden layers, the accuracy converged faster to values close to 100%. The worst accuracy using ANN was for samples on EDTA tubes, using 1 and 2 HL and 100 epochs with a value of 85%; this improved to reaching 91% with 2 HL and 300 epochs. For the samples in lithium heparin tubes, all accuracy values were between 97% and 99% within both input dataset. Data balancing improved accuracy slightly for samples contained in EDTA tubes; for samples stored in lithium heparin tubes, there was no significant improvement. When using random forest models, the high accuracy obtained for the unbalanced data was remarkable, being the best for the case of SF in EDTA tubes. Again, data balancing slightly improved the accuracy. The SVM models were found to have low accuracy, having the lowest accuracy of all the models compared.

**Figure 7:**
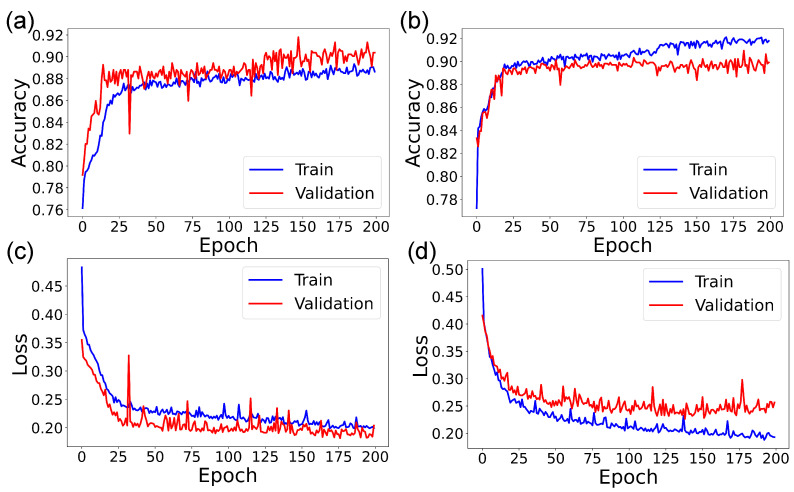
Accuracy and loss curves for SF in tubes with EDTA. ANN with 2 hidden layers and 200 epochs. (**a**) Accuracy for unbalanced data. (**b**) Accuracy for balanced data. (**c**) Loss for unbalanced data. (**d**) Loss for balanced data.

**Figure 8:**
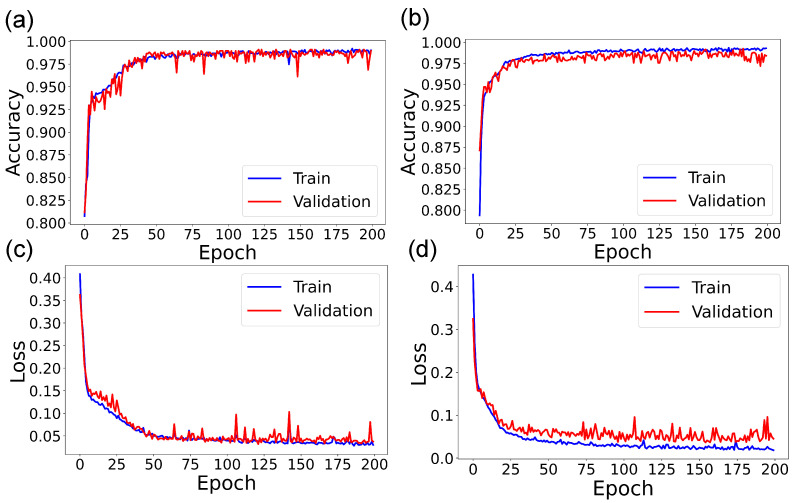
Accuracy and loss curves for SF in tubes with Lithium heparin. ANN with 2 hidden layers and 200 epochs. (**a**) Accuracy for unbalanced data. (**b**) Accuracy for balanced data. (**c**) Loss for unbalanced data. (**d**) Loss for balanced data.

Figure 7 and Figure 8 show the accuracy and loss curves when the ANN was trained for the HL = 2 and epoch = 200 cases. The blue curve shows the training data’s progression, and the red one shows the progression of the validation data. Curves are shown for unbalanced and balanced data for each type of container. It can be seen that, for the case of samples in EDTA tubes and unbalanced data, the accuracy reached 88% at around 75 epochs, and when balancing the data, the accuracy was 90% at about 25 epochs. For samples stored in tubes with lithium heparin, the accuracy reached 95% in 25 epochs, and when balancing the data, in less than 25 epochs.

Table 8 and Table 9 bring together the confusion matrices for each scenario. Note that the TP and TN parameters are shaded and follow the distribution in Figure 6. As can be seen, the FN and FP parameters for the cases with higher accuracy tended to 0.

## 4. Conclusions

This work showed that the technique used to characterize the viscous properties of SF using a QCR-based sensor could help classify and differentiate infectious SF from other nosological entities. The results are encouraging; however, a more extensive study is needed. We have shown an ANN that aids in the classification of inflammatory and infectious SF using data associated with the viscous properties of SF obtained using a QCR sensor. The extraordinary ability of AI technologies to classify data in a way that is superior to conventional techniques was demonstrated. In the comparison carried out in this work, the improvements through the use of classification models such as random forests and neural networks were noticeable. When comparing both classifications of SF using Δf, ΔΓ, and η individually, there were some statistically significant differences. Still, they did not perform well on their own in classification. However, high accuracy was obtained by training an ANN to differentiate between two types of SF. We achieved higher precision values for samples stored in tubes with lithium heparin. With the results obtained, developing a sensor using QCR for SF classification is promising. However, it is necessary to continue increasing the amount of information obtained with the sensor by measuring more samples and extending its application to other types of biological fluids. The proposed technique presents a novel method for the classification of human fluids. The advantages are: (i) the use of a low sample volume (50 μL), (ii) the low cost of the device, and (iii) portability. This makes it accessible to any laboratory and should promote interest in further development. As future work, the dataset could be further augmented, and a comparison between different classification models can be performed.

## Figures and Tables

**Table 1:** SF samples.

	EDTA	Lithium Heparin
Inflammatory	25	21
Infectious	8	7
Total	33	28

**Table 2:** ANN features.

Parameter	Features
Input Layer	Neurons: 3 (or 5) Activation function: Relu
Hidden Layers	1, 2 Neurons: 50 Activation function: Relu
Output Layer	Neurons: 2 Activation function: Softmax
Training Epochs	100, 200, 300
Batch size	16
Optimizer Type	Adam

**Table 3:** Comparison between mean values for SF contained in tubes with EDTA.

Age (yr)	55.52 ± 27.53	72.75 ± 15.27	0.08
WBC (/mm${}^{3}$)	9060 ± 12,526	52,575.62 ± 75,126.19	0.02
Neutrophils (per)	57.28 ± 36.39	85.50 ± 12.43	0.02
Glucose (mg/dL)	99.23 ± 32.11	64.37 ± 35.97	0.05
Proteins (g/dL)	3.87 ± 0.82	4.15 ± 0.49	0.23
Δf (Hz)	−3665.36 ± 135.34	−3675.87 ± 104.57	0.25
ΔΓ (Hz)	1787.47 ± 66.97	1810.47 ± 53.34	0.04
η (mPa· s)	3.46 ± 0.21	3.43 ± 0.30	0.11

**Table 4:** Comparison between mean values for SF contained in tubes with lithium heparin.

Age (yr)	64.66 ± 18.96	71.85 ± 16.27	0.29
WBC (/mm${}^{3}$)	9032.76 ± 13,478.73	57,789.28 ± 79,560.83	0.03
Neutrophils (%)	63.11 ± 36.80	84.00 ± 12.62	0.16
Glucose (mg/dL)	99.23 ± 32.11	59.57 ± 35.98	0.01
Proteins (g/dL)	3.87 ± 0.82	4.11 ± 0.52	0.29
Δf (Hz)	−3775.40 ± 106.55	−3812.91 ± 109.05	0.03
ΔΓ (Hz)	1861.21 ± 95.89	1908.10 ± 72.09	0.01
η (mPa· s)	3.76 ± 0.31	3.67 ± 0.18	0.13

**Table 5:** Area under the ROC curve values for the parameters as predictors for infectious fluid (SF in tubes with EDTA).

WBC (/mm${}^{3}$) [10]	1.00	1.00–1.00	0.00
PCT serum [10]	0.82	0.71–0.92	0.05
PCT SF [10]	0.65	0.51–0.78	0.06
WBC (/mm)	0.78	0.60–0.97	0.09
Neutrophils (%)	0.76	0.58–0.94	0.09
Glucose (mg/dL)	0.26	0.03–0.49	0.12
Proteins (g/dL)	0.64	0.44–0.85	0.10
Δf (Hz)	0.55	0.46–0.65	0.04
ΔΓ (Hz)	0.60	0.51–0.69	0.04
η (mPa· s)	0.42	0.31–0.52	0.05

**Table 6:** Area under the ROC curve values for the parameters as predictors for infectious fluid (SF in tubes with lithium heparin).

WBC (/mm${}^{3}$) [10]	1.00	1.00–1.00	0.00
PCT serum [10]	0.82	0.71–0.92	0.05
PCT SF [10]	0.65	0.51–0.78	0.06
WBC (/mm)	0.8	0.61–0.99	0.09
Neutrophils (%)	0.68	0.46–0.91	0.11
Glucose (mg/dL)	0.20	0.00–0.43	0.11
Proteins (g/dL)	0.62	0.39–0.85	0.11
Δf (Hz)	0.61	0.51–0.72	0.05
ΔΓ (Hz)	0.65	0.55–0.74	0.04
η (mPa· s)	0.42	0.33–0.50	0.04

**Table 7:** Accuracy obtained for each ANN scenario, including the values obtained for the SVM and RF models.

Model	EDTA		Lithium Heparin	
	Data	B. Data	Data	B. Data
ANN; HL: 1; Epochs: 100	0.85	0.90	0.98	0.97
ANN; HL: 1; Epochs: 200	0.88	0.91	0.98	0.98
ANN; HL: 1; Epochs: 300	0.90	0.91	0.99	0.98
ANN; HL: 2; Epochs: 100	0.87	0.91	0.97	0.98
ANN; HL: 2; Epochs: 200	0.88	0.92	0.98	0.97
ANN; HL: 2; Epochs: 300	0.91	0.91	0.98	0.98
SVM	0.79	0.76	0.87	0.69
RF	0.91	0.97	0.96	0.98

**Table 8:** Confusion matrix for each scenario. SF in tubes with EDTA.

	ANN Setting	Data		Balanced Data	
EDTA	HL: 1 Epochs: 100	488	86	485	99
		20	152	05	553
	HL: 1 Epochs: 200	514	60	593	91
		29	143	09	549
	HL: 1 Epochs: 300	541	33	501	83
		35	137	11	547
	HL: 2 Epochs: 100	504	70	496	88
		21	151	07	551
	HL: 2 Epochs: 200	511	63	516	68
		26	146	23	535
	HL: 2 Epochs: 300	562	12	506	78
		53	119	19	539

**Table 9:** Confusion matrix for each scenario. SF in tubes with lithium heparin.

	ANN Setting	Data		Balanced Data	
Lithium heparin	HL: 1 Epochs: 100	627	04	586	10
		10	147	22	623
	HL: 1 Epochs: 200	626	05	580	16
		07	150	08	637
	HL: 1 Epochs: 300	629	02	590	06
		05	152	11	634
	HL: 2 Epochs: 100	615	16	589	07
		05	152	09	636
	HL: 2 Epochs: 200	626	05	590	06
		06	151	20	625
	HL: 2 Epochs: 300	628	03	586	10
		06	151	05	640

## Data Availability

The data presented in this study are available on request from the corresponding author.

## References

[1] Mundt A.L., Shanahan K. (2010). Graff’s Textbook of Routine Urinalysis and Body Fluids.

[2] Damiano J., Bardin T. (2004). Synovial fluid. EMC-Rhumatologie-Orthopedie.

[3] Brannan S.R., Jerrard D.A. (2006). Synovial fluid analysis. J. Emerg. Med..

[4] Martínez-Castillo A., Núñez C., Cabiedes J. (2010). Synovial fluid analysis. Reumatología Clínica (Engl. Ed.).

[5] Stafford C.T., Niedermeier W., Holley H.L., Pigman W. (1964). Studies on the concentration and intrinsic viscosity of hyaluronic acid in synovial fluids of patients with rheumatic diseases. Ann. Rheum. Dis..

[6] Rojas C. (2012). Estudio del líquido sinovial. Guía de Procedimientos en Reumatología.

[7] West S.G. (2014). Rheumatology Secrets.

[8] Swan A., Amer H., Dieppe P. (2002). The value of synovial fluid assays in the diagnosis of joint disease: A literature survey. Ann. Rheum. Dis..

[9] Sangha O. (2000). Epidemiology of rheumatic diseases. Rheumatology.

[10] Talebi-Taher M., Shirani F., Nikanjam N., Shekarabi M. (2013). Septic versus inflammatory arthritis: Discriminating the ability of serum inflammatory markers. Rheumatol. Int..

[11] Miranda-Martínez A., Rivera-González M.X., Zeinoun M., Carvajal-Ahumada L.A., Serrano-Olmedo J.J. (2021). Viscosity measurement sensor: A prototype for a novel medical diagnostic method based on quartz crystal resonator. Sensors.

[12] Ahumada L.A.C., González M.X.R., Sandoval O.L.H., Olmedo J.J.S. (2016). Evaluation of hyaluronic acid dilutions at different concentrations using a quartz crystal resonator (QCR) for the potential diagnosis of arthritic diseases. Sensors.

[13] Tan F., Qiu D.Y., Guo L.P., Ye P., Zeng H., Jiang J., Tang Y., Zhang Y.C. (2016). Separate density and viscosity measurements of unknown liquid using quartz crystal microbalance. AIP Adv..

[14] Huang X., Bai Q., Hu J., Hou D. (2017). A practical model of quartz crystal microbalance in actual applications. Sensors.

[15] Cao-Paz A.M., Rodríguez-Pardo L., Fariña J., Marcos-Acevedo J. (2012). Resolution in QCM sensors for the viscosity and density of liquids: Application to lead acid batteries. Sensors.

[16] Fort A., Panzardi E., Vignoli V., Tani M., Landi E., Mugnaini M., Vaccarella P. (2021). An adaptive measurement system for the simultaneous evaluation of frequency shift and series resistance of QCM in liquid. Sensors.

[17] Hai W., Goda T., Takeuchi H., Yamaoka S., Horiguchi Y., Matsumoto A., Miyahara Y. (2017). Specific Recognition of Human Influenza Virus with PEDOT Bearing Sialic Acid-Terminated Trisaccharides. ACS Appl. Mater. Interfaces.

[18] Wang R., Wang L., Callaway Z.T., Lu H., Huang T.J., Li Y. (2017). A nanowell-based QCM aptasensor for rapid and sensitive detection of avian influenza virus. Sens. Actuators B Chem..

[19] Kim Y.K., Lim S.I., Cho Y.Y., Choi S., Song J.Y., An D.J. (2014). Detection of H3N2 canine influenza virus using a Quartz Crystal Microbalance. J. Virol. Methods.

[20] Lim H.J., Saha T., Tey B.T., Tan W.S., Ooi C.W. (2020). Quartz crystal microbalance-based biosensors as rapid diagnostic devices for infectious diseases. Biosens. Bioelectron..

[21] Wangmaung N., Chomean S., Promptmas C., Mas-oodi S., Tanyong D., Ittarat W. (2014). Silver quartz crystal microbalance for differential diagnosis of Plasmodium falciparum and Plasmodium vivax in single and mixed infection. Biosens. Bioelectron..

[22] Ly T.N., Park S., Park S.J. (2016). Detection of HIV-1 antigen by quartz crystal microbalance using gold nanoparticles. Sens. Actuators B Chem..

[23] He F., Zhang L. (2002). Rapid diagnosis of M. tuberculosis using a piezoelectric immunosensor. Anal. Sci..

[24] He F., Zhang L., Zhao J., Hu B., Lei J. (2002). A TSM immunosensor for detection of M. tuberculosis with a new membrane material. Sens. Actuators B Chem..

[25] Hiatt L.A., Cliffel D.E. (2012). Real-time recognition of Mycobacterium tuberculosis and lipoarabinomannan using the quartz crystal microbalance. Sens. Actuators B Chem..

[26] Wang Y., Moss M.A. (2016). Effect of Resveratrol and Derivatives on Interactions between Alzheimer’s Disease Associated A*β* Protein Oligomers and Lipid Membranes: A Quartz Crystal Microbalance Analysis. Biophys. J..

[27] Hwang S.S., Chan H., Sorci M., Van Deventer J., Wittrup D., Belfort G., Walt D. (2019). Detection of amyloid *β* oligomers toward early diagnosis of Alzheimer’s disease. Anal. Biochem..

[28] Yılmaz M., Bakhshpour M., Göktürk I., Pişkin A.K., Denizli A. (2021). Quartz Crystal Microbalance (QCM) Based Biosensor Functionalized by HER2/neu Antibody for Breast Cancer Cell Detection. Chemosensors.

[29] Liao S., Ye P., Chen C., Zhang J., Xu L., Tan F. (2022). Comparing of Frequency Shift and Impedance Analysis Method Based on QCM Sensor for Measuring the Blood Viscosity. Sensors.

[30] Miranda-Martínez A., Yan H., Silveira V., Serrano-Olmedo J.J., Crouzier T. (2022). Portable Quartz Crystal Resonator Sensor for Characterising the Gelation Kinetics and Viscoelastic Properties of Hydrogels. Gels.

[31] Nayak R., Jain L.C., Ting B.K.H. (2001). Artificial neural networks in biomedical engineering: A review. Computational Mechanics–New Frontiers for the New Millennium.

[32] Haglin J.M., Jimenez G., Eltorai A.E.M. (2019). Artificial neural networks in medicine. Health Technol..

[33] Mohan Y., Chee S.S., Xin D.K.P., Foong L.P. Artificial neural network for classification of depressive and normal in EEG. Proceedings of the 2016 IEEE EMBS Conference on Biomedical Engineering and Sciences (IECBES).

[34] Olaniyi E.O., Oyedotun O.K., Helwan A., Adnan K. Neural network diagnosis of heart disease. Proceedings of the 2015 International Conference on Advances in Biomedical Engineering (ICABME).

[35] Adak M.F., Lieberzeit P., Jarujamrus P., Yumusak N. (2020). Classification of alcohols obtained by QCM sensors with different characteristics using ABC based neural network. Eng. Sci. Technol. Int. J..

[36] Mumyakmaz B., Özmen A., Ebeoğlu M.A., Taşaltın C., Gürol İ. (2010). A study on the development of a compensation method for humidity effect in QCM sensor responses. Sens. Actuators B Chem..

[37] Reznik A.M., Galinskaya A.A., Dekhtyarenko O.K., Nowicki D.W. (2005). Preprocessing of matrix QCM sensors data for the classification by means of neural network. Sens. Actuators B Chem..

[38] Osisanwo F., Akinsola J., Awodele O., Hinmikaiye J., Olakanmi O., Akinjobi J. (2017). Supervised machine learning algorithms: Classification and comparison. Int. J. Comput. Trends Technol. (IJCTT).

[39] Azar A.T., Elshazly H.I., Hassanien A.E., Elkorany A.M. (2014). A random forest classifier for lymph diseases. Comput. Methods Programs Biomed..

[40] Keiji Kanazawa K., Gordon J.G. (1985). The oscillation frequency of a quartz resonator in contact with liquid. Anal. Chim. Acta.

[41] Johannsmann D. (2015). The quartz crystal microbalance in soft matter research. Soft Biol. Matter.

[42] Lee C.P., Lin C.J. (2013). A study on L2-loss (squared hinge-loss) multiclass SVM. Neural Comput..

[43] Ciepliński M., Kasprzak M., Grandtke M., Steliga A., Kamiński P., Jerzak L. (2019). The effect of dipotassium EDTA and lithium heparin on hematologic values of farmed brown trout Salmo trutta (L.) spawners. Aquac. Int..

[44] Baien S.H., Langer M.N., Heppelmann M., von Köckritz-Blickwede M., De Buhr N. (2018). Comparison between K3EDTA and lithium heparin as anticoagulant to isolate bovine granulocytes from blood. Front. Immunol..

